# THYLENETETRAHYDROFOLATE REDUCTASE GENE POLYMORPHISMS AND SUSCEPTIBILITY TO ESOPHAGEAL CANCER: A CASE-CONTROL STUDY

**DOI:** 10.1590/0102-672020220002e1684

**Published:** 2022-09-09

**Authors:** Evelise Pelegrinelli Zaidan, Michele Tatiana Pereira Tomitão, Marina Alessandra Pereira, Marcia Saldanha Kubrusly, Adriana Vaz Safatle-Ribeiro, Flavio Roberto Takeda, Ivan Cecconello, Ulysses Ribeiro

**Affiliations:** 1 Universidade de São Paulo, Faculty of Medicine, Cancer Institute, University Hospital, Department of Gastroenterology – São Paulo (SP), Brazil.

**Keywords:** Esophageal Neoplasms, Adenocarcinoma, Carcinoma, Squamous Cell, Polymorphism, Genetic, Methylenetetrahydrofolate Reductase (NADPH2), Neoplasias Esofágicas, Adenocarcinoma, Carcinoma de Células Escamosas, Polimorfismo Genético, Metilenotetra-Hidrofolato Redutase (NADPH2)

## Abstract

**BACKGROUND:**

The enzyme methylenetetrahydrofolate reductase is engaged in DNA synthesis through folate metabolism. Inhibiting the activity of this enzyme increases the susceptibility to mutations, and damage and aberrant DNA methylation, which alters the gene expression of tumor suppressors and proto-oncogenes, potential risk factors for esophageal cancer.

**AIMS:**

This study aimed to investigate the association between methylenetetrahydrofolate reductase 677C>T and methylenetetrahydrofolate reductase 1298A>C polymorphisms and susceptibility to esophageal cancer, by assessing the distribution of genotypes and haplotypes between cases and controls, as well as to investigate the association of polymorphisms with clinical and epidemiological characteristics and survival.

**METHODS:**

A total of 109 esophageal cancer patients who underwent esophagectomy were evaluated, while 102 subjects constitute the control group. Genomic DNA was isolated from the peripheral blood buffy coat followed by amplification by polymerase chain reaction and real-time analysis. Logistic regression was used to assess associations between polymorphisms and the risk of developing esophageal cancer.

**RESULTS:**

There was no association for methylenetetrahydrofolate reductase 677C>T and methylenetetrahydrofolate reductase 1298A>C polymorphisms and haplotypes, with esophageal cancer susceptibility. Esophageal cancer patients carrying methylenetetrahydrofolate reductase 677TT polymorphism had higher risk of death from the disease. For polymorphic homozygote TT genotype, the risk of death significantly increased compared to wild-type genotype methylenetetrahydrofolate reductase 677CC (reference) cases (p=0.045; RR=2.22, 95%CI 1.02–4.83).

**CONCLUSIONS:**

There was no association between methylenetetrahydrofolate reductase 677C>T and methylenetetrahydrofolate reductase 1298A>C polymorphisms and esophageal cancer susceptibility risk. Polymorphic homozygote genotype methylenetetrahydrofolate reductase 677TT was associated with higher risk of death after surgical treatment for esophageal cancer.

## RESUMO

**RACIONAL:**

A enzima metilenotetrahidrofolato redutase está envolvida na síntese de DNA através do metabolismo do folato. A inibição da sua atividade aumenta a suscetibilidade a mutações, danos e metilação aberrante do DNA, o que altera a expressão gênica de supressores tumorais e proto-oncogenes, potenciais fatores de risco para câncer de esôfago.

**OBJETIVOS:**

Investigar a associação entre os polimorfismos metilenotetrahidrofolato redutase 677C>T e metilenotetrahidrofolato redutase 1298A>C e a suscetibilidade ao câncer de esôfago, avaliando a distribuição de genótipos e haplótipos entre casos e controles, bem como investigar a associação de polimorfismos com características clínicas, epidemiológicas e sobrevida.

**MÉTODOS:**

Avaliaram-se 109 pacientes com câncer de esôfago submetidos à esofagectomia, enquanto 102 indivíduos constituaram o grupo controle. O DNA genômico do sangue periférico foi isolado e submetido à amplificação por reação em cadeia da polimerase em tempo real. A associação entre os polimorfismos e o risco de desenvolver câncer de esôfago foi avaliada por regressão logística.

**RESULTADOS:**

Não houve associação dos polimorfismos e haplótipos metilenotetrahidrofolato redutase 677C>T e metilenotetrahidrofolato redutase 1298A>C com a suscetibilidade ao câncer de esôfago. Pacientes com câncer de esôfago portadores do polimorfismo metilenotetrahidrofolato redutase 677TT apresentaram maior risco de morte pela doença. Para o genótipo TT homozigoto polimórfico, o risco de morte aumentou significativamente em comparação com os casos do genótipo selvagem metilenotetrahidrofolato redutase 677CC (referência) (p=0,045; RR=2,22, IC95% 1,02–4,83).

**CONCLUSÕES:**

Não houve associação entre os polimorfismos metilenotetrahidrofolato redutase 677C>T e metilenotetrahidrofolato redutase 1298A>C e o risco de suscetibilidade ao câncer de esôfago. O genótipo homozigoto polimórfico metilenotetrahidrofolato redutase 677TT associou-se a um maior risco de óbito após tratamento cirúrgico para câncer de esôfago.

## INTRODUCTION

Esophageal cancer corresponds to the eighth leading cause of cancer worldwide and affects more than 450,000 people yearly. The 5-year survival rate is low, generally around 10%, while the mortality rate is similar to the incidence, due to its high lethality (0.88)^11,24^. Lymph node metastasis, followed by its rapid progression, explains the poor prognosis of this disease^1,21 25^.

Besides hereditary mechanisms, life habits and the environment can induce or perhaps prevent carcinogenesis of the esophagus. Interactive effects on gene and environmental factors may also vary among different regions of the world^5,21,26^. Diet low in folate, food intake at high temperatures, chronic mucosal irritation, and especially the consumption of alcohol and tobacco have been described as risk factors for esophageal cancer^11,13,15^. Consumption of alcohol and tobacco is responsible for 45% new cases of esophageal cancer in men and 11% in women, with a synergistic effect^10,19,22^.The transformation of the malignant phenotype by sequential order of mutations has multiple causes; however, it is poorly defined. Gene expression varies in different stages from normal mucosa to metastatic tumors^6,26^. Furthermore, DNA methylation has important effects on cell development and function. This epigenetic modification, including the genomic hypomethylation and regional hypermethylation, changes the genomic stability and gene expression of tumor suppressors and proto-oncogenes, common event in carcinogenesis^7,8,18,20,27^. The enzyme methylenetetrahydrofolate reductase (MTHFR) plays an important role in folate metabolism. The substrate for the MTHFR, i.e., 5,10-methylenetetrahydrofolate enzyme (THF), is the methyl group donor for the conversion of deoxyuridylate (dUMP) to deoxythymidylate (dTMP) required for DNA synthesis^7,9,17,27^. Folate deficiency and the change in MTHFR activity have been described as risk factors for esophageal cancer through the following two mechanisms: one is the erroneous incorporation of uracil in DNA, which leads to disruption of its integrity and repair, increasing the susceptibility to mutations and DNA damage^12,18,23,27^. The other mechanism is aberrant DNA methylation, which occurs almost exclusively at CpG dinucleotide (CpG islands) at the 5′ position of the cytosine ring to form 5-methylcytosine generally protected from methylation in normal tissues^6,8^.

The MTHFR gene contains a coding region with 11 exons and is located on chromosome 1p36.3. Approximately 60 polymorphisms have been described in this gene. The C677T and A1298C polymorphism genotypes variants of MTHFR gene have been associated with significant reductions in enzymatic activity and consequently interruption of folate metabolism^20^.

The C677T variant (rs1801133) is a C to T transition at nucleotide 677 in exon 4, resulting in the conversion of alanine to valine at position 222 of the amino acid sequence^7,13,27^. This polymorphism is associated with reduced enzyme activity, limiting the conversion of 5,10-methylenetetrahydrofolate to 5-MTHFR^9^.

Compared to homozygous wild subtype (CC), the polymorphic heterozygous (CT) expresses the enzyme with 65% of activity since the polymorphic homozygote (TT) expresses the enzyme with 30% of activity. Both are associated with lower serum folate levels and higher levels of plasma homocysteine, with consequent hypomethylation of DNA, which is a relevant factor of carcinogenesis^9,13,27^.

Another polymorphism that alters the activity of MTHFR is the substitution of A for C at nucleotide 1298 (rs1801131). This results in a change of the amino acid alanine for glutamate at exon 7, which reduces enzyme activity. This variant is less severe than 677^7^. Subjects with CC genotype at residue 1298 have 60% of the enzymatic capacity compared to the AA genotype^4,7^.

The purpose of this study was to investigate the association between MTHFR 677C>T and MTHFR 1298A>C and the susceptibility to esophageal cancer, by assessing the distribution of genotypes and haplotypes between cases and controls, as well as to investigate the association of such polymorphisms with clinical and epidemiological characteristics and survival outcomes.

## METHODS

We conducted a unicenter case-control study of esophageal cancer in the Brazilian population. A total of 109 patients with a histologically confirmed diagnosis of esophageal cancer who underwent potentially curative esophagectomy at the University Hospital, from June 2001 to January 2008, were enrolled. Exclusion criterion for patients with cancer was neoadjuvant therapy or previous esophageal surgical intervention.

The control group consisted of 102 patients who were operated on for benign disease at the University Hospital. Inclusion criteria for the control group were as follows: no personal or familial cancer history, no diseases associated with known risk factors for developing esophageal cancer, or diseases associated with tobacco and alcohol, Barrett’s esophagus, chronic reflux esophagitis, and esophageal achalasia. The case and control groups were matched for gender, age, and ethnicity.

The clinicopathological data (e.g., age, gender, ethnicity, smoking, alcohol intake, histological type, and tumor stage) were collected through questionnaires given to research participants and revised according to the information from the medical hospital records. Due to the small number of Asiatics, Indians, Mulattos, and African Americans, the patients were grouped into white and non-white. The stage of the disease was classified according to the American Joint Committee on Cancer and the International Union Against Cancer (AJCC/UICC) guidelines^1^.

The genomic DNA was isolated from peripheral blood buffy coat using the extraction and purification kit PureLinkT Genomic DNA Mini Kit (Invitrogen-Life Technologies, Carlsbad, CA, USA) according to manufacturer’s instructions. The basis of the identification technique utilized probes labeled with fluorophores TaqMan® SNP Genotyping Assays (Applied Biosystems, Foster City, CA, USA), amplification by polymerase chain reaction (PCR), and real-time analysis.

This study was approved by the Ethics Committee for Research Project Analysis (CAPPesq), University Hospital of Faculty of Medicine of Universidade de São Paulo, under 9504 online registration, and in conformance with applicable regulatory requirement and Good Clinical Practice (GCP) (number: 05176812.2.0000.0068).

### Statistical analysis

The associations between polymorphisms in both groups were analyzed using Fisher’s exact test. Logistic regression was used to quantify these associations in terms of odds ratios with their respective 95% confidence intervals (CI). Frequencies and percentages were calculated for variables, and Student’s t-test and Pearson’s chi-square test were used to analyze continuous and categorical data. Survival curves were built using Kaplan-Meier method, and the differences between the curves were examined by the log-rank test. The follow-up time was considered from the date of surgery to the date of death or last medical appointment. Simple Cox regression was applied to calculate the risk of death according to the genotypes. The analyses were carried out through the R program, version 3.1.1 (R Core Team, 2014). The frequency of haplotypes was calculated from the EM algorithm (Expecting-Maximization) and compared by chi-square test, as well as the evaluation of the unbalance of Hardy-Weinberg equilibrium^2^. A p-value <0.05 was considered statistically significant.

## RESULTS

Table 1 shows the distribution of demographic and clinical data of 109 patients with esophageal cancer and 102 controls. There were no differences between groups regarding age, gender, and ethnicity.

**Table 1:** Distribution of demographic and clinical data of esophageal cancer patients (n=109) and control (n=102).

Variable		Total	Case	Control	p-value
		n=211 (%)	n=109 (%)	n=102 (%)	
Age					0.102
	Mean (SD)	59.33±12.17	60.66±10.54	57.89±13.60	
	Median	59	59	58	
Gender					0.473
	Female	37 (17.5)	17 (15.6)	20 (19.6)	
	Male	174 (82.4)	92 (84.4)	82 (80.3)	
Ethnicity					0.210
	White	157 (74.4)	77 (70.6)	80 (78.4)	
	Non-white	54 (25.5)	32 (29.3)	22 (21.5)	
Alcohol intake*					0.001
	Nondrinkers	84 (80.7)	18 (17.6)	66 (65.7)	
	Ex-drinkers	63 (60.5)	49 (48.0)	14 (13.7)	
	Drinkers	57 (54.8)	35 (34.3)	22 (21.5)	
Smoking*					0.001
	Nonsmokers	59 (56.7)	14 (13.8)	45 (44.1)	
	Ex-smokers	77 (74.0)	41 (40.5)	36 (35.2)	
	Smokers	67 (64.4)	46 (45.5)	21 (20.5)	

SD: standard deviation.

* Missing data variables: alcohol intake seven cases; Smoking: eight cases. Bold denotes statistically significant p-values.

Ex-alcoholic group (48.0%) and alcohol consumers (34.3%) were more prevalent among patients with esophageal cancer (case group) (p=0.001). In addition, ex-smokers (40.5%) and active smokers (45.5%) were also more frequent among patients with esophageal cancer (p=0.001).

Among the 109 patients in control group, 43 (39.5%) were adenocarcinomas and 66 (60.5%) were histologically classified as squamous cells carcinoma. Regarding the pathological staging (pTNM), 10.6% of the patients were classified as stage I, 35.9% as stage II, 45.6% as stage III, and 7.7% as stage IV.

The distribution of genotypes in both groups, cases and controls, is described in Table 2 according to Hardy-Weinberg principle.

**Table 2:** Distribution of genotypes of esophageal cancer patients and control according to Hardy-Weinberg principle.

Polymorphism		Case	Hardy-Weinberg (p-value)	Total
			Control	
MTHFR 677C>T		0.679	1,000	0.666
	(rs1801133)			
MTHFR 1298A>C		**0.016**	0.806	0.183
	(rs1801131)			

Bold denotes statistically significant p-values.

There was no significant association for the MTHFR 677C>T and MTHFR 1298A>C polymorphisms and esophageal cancer susceptibility (Table 3).

**Table 3:** Distribution of genotypes frequencies and odds ratio for esophageal cancer patients and control.

Genotype (wild)		Polymorfisms	Total	Case	Control	p-value	OR	95%CI	95%CI	p-value
			n=211 (%)	n=109 (%)	n=102 (%)					
MTHFR 677C>T		CC	87 (41.23)	46 (42.2)	41 (40.2)	0.963	Reference			
	(rs1801133)	CT	95 (45.02)	48 (44.0)	47 (46.1)		0.95	0.46–1.96	0.46–1.96	0.885
		TT	29 (13.74)	15 (13.7)	14 (13.7)		1.12	0.4–3.15	0.4–3.15	0.837
MTHFR 1298A>C		AA	115 (54.5)	63 (57.8)	52 (50.9)	0.140	Reference			
	(rs1801131)	AC	76 (36.0)	33 (30.3)	43 (42.1)		0.63	0.3–1.32	0.3–1.32	0.216
		CC	20 (9.5)	13 (11.9)	7 (6.8)		2.59	0.7–9.61	0.7–9.61	0.156
			Total	Case	Control					
Genotype (wild and polymorphic)			n=211 (%)	n=109 (%)	n=102 (%)	p-value	OR	95%CI	95%CI	p-value
MTHFR 677C>T		CC	87 (41.23)	46 (42.2)	41 (40.2)	0.780	Reference			
	(rs1801133)	CT+TT	124 (58.76)	63 (57.8)	61 (59.8)		0.99	0.5–1.97	0.5–1.97	0.971
MTHFR 1298A>C						0.330				
	(rs1801131)	AA	115 (54.5)	63 (57.8)	52 (50.9)		Reference	0.42–1.64		
		AC+CC	46	46 (42.2)	50 (49.02)		0.83	0.42–1.64	0.42–1.64	0.592

OR: odds ratio; CI: confidence interval; CC: homozygous wild subtype; CT: polymorphic heterozygous; TT: polymorphic homozygote; A, C, T: polymorfisms.

To examine the association of the haplotypes and esophageal cancer, a comparison of the frequencies of haplotypic blocks formed by MTHFR polymorphisms between cases and controls was performed. There was no statistical difference in the evaluation of haplotype blocks AC (p=0.76), AT (p=0.81), and CC (p=0.96) formed by the MTHFR 677C>T and MTHFR 1298A>C polymorphism between the two groups and also no differences regarding histological type or other clinicopathological characteristics.

### Overall survival analysis

The median overall survival of esophageal cancer patients was 23 months, and 62 (56.8%) patients died due to esophageal cancer. Regarding the pTNM staging (grouped into stages I/II and stages III/IV), there were statistically significant differences in survival; patients with more advanced disease had a worse overall survival (p=0.01).

Multiple Cox logistic regression, adjusted for age, histological type, gender, ethnicity, smoking, and alcohol consumption, showed a statistically significant difference for the MTHFR 677C>T polymorphism. For polymorphic homozygote TT genotype patients, the risk of death increased by 2.22 times, greater than for the wild-type genotype MTHFR 677CC (reference) patients (p=0.045; RR=2.22, 95%CI 1.02–4.83) (Table 4).

**Table 4:** Relative risk of death related to genotypes. Multiple Cox regression analysis adjusted by age, histological type, gender, ethnicity, alcohol intake, and smoking variables.

Genotypes		Polymorfisms	RR	95%CI	p-value	Genotypes	RR	95%CI	p-value
MTHFR 677C>T									
	(rs1801133)	CC	Reference			CC	Reference		
		CT	1.15	0.64–2.06	0.643	CT+TT	1.35	0.79–2.31	0.272
		TT	2.22	1.02–4.83	0.045				
MTHFR 1298A>C									
	(rs1801131)	AA	Reference			AA	Reference		
		AC	1	0.52–1.95	0.992	AC+CC	0.79	0.43–1.45	0.451
		CC	0.5	0.2–1.27	0.145				

RR: relative risk; CI: confidence interval; CC: homozygous wild subtype; CT: polymorphic heterozygous; TT: polymorphic homozygote; A, C, T: polymorfisms.

**Figure 1:**
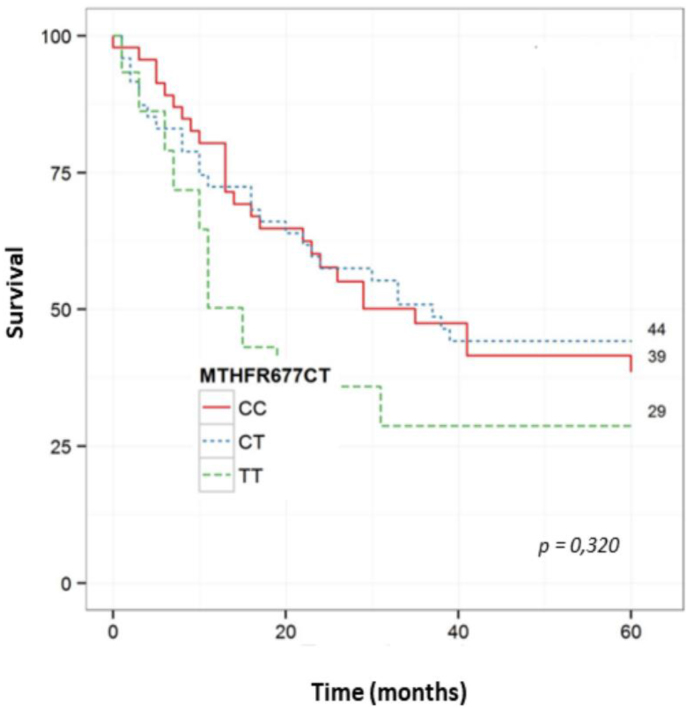
Overall survival of patients with esophageal cancer according to the genotypes – case group (n=109) (p=0.32).

The survival curve using the Kaplan-Meier method and log-rank test, comparing of the three genotypes of the polymorphism MTHFR 677C>T, demonstrated no statistical difference in the overall survival for esophageal cancer patients between the groups (p=0.32) (Figure 1).

## DISCUSSION

In this study, MTHFR 677C>T and MTHFR 1298A>C polymorphisms, which are variants of MTHFR gene, were not associated with high susceptibility risk for esophageal cancer. Nonetheless, homozygous MTHFR 677 TT was related to a more aggressive disease.

Folate deficiency and changing the MTHFR enzyme activity have been associated with the increased risk of esophageal cancer by the increased susceptibility to mutations, and damage and aberrant DNA methylation^12,18,23,27^. Polymorphic genes may be involved in the metabolism of certain foods or toxins, inflammatory responses, and drug interaction, among others^3^.

The selected polymorphisms for analysis in this study, i.e. MTHFR 677C>T and MTHFR 1298A>C, are located in the coding region of the gene and are associated with significant reduction in enzyme activity^20^.

The frequency of the MTHFR 677C>T polymorphism genotypes in esophageal cancer patients in this study was CC 42.2%, CT 44.0%, and TT 13.7%. According to previous reports, the frequency of C677T alleles has shown a variation between different ethnic groups. In China, the frequency between subjects with esophageal cancer is CC 18.3%, CT 42.9%, and TT 38.8%^23^. In Japan, genotype frequency distribution has been similar to this study, with CC 38.2%, CT 49.7%, and TT 12.1%^27^. In contrast, among North Americans with esophageal cancer, the TT genotype frequency has been as high as 65%^14^.

Regarding the polymorphism of MTHFR 1298A>C, the genotype distribution frequencies in this study was AA 57.8%, AC 30.2%, and CC 11.93%, findings quite different when compared to Japanese subjects with esophageal cancer, with AA 74.3%, AC 24.2%, and CC 1.4%^23^. Among North Americans, the genotype frequency for CC was 12.9%, similar to this study^14^.

In this research, no significant difference was observed in the genotype frequency distribution, alleles, or haplotypes between cases and controls for the MTHFR 677C>T and MTHFR 1298A>C polymorphisms^18^. No relationship has also been observed between MTHFR 677C>T polymorphism and esophageal cancer in Chinese population; however, in some studies, there was a significant association between MTHFR 677TT genotype and esophageal cancer, suggesting that the effect of this polymorphism may vary according to ethnicity^14,20,23^. No relationship between MTHFR 1298A>C polymorphism and esophageal cancer susceptibility has been described, as demonstrated in this study^14,18,20,23^.

Discrepancies found between the results in several studies suggested that the presence of other factors could modulate the polymorphism behavior, including exposure to alcohol, tobacco, anti-inflammatory drugs, and vast genetic variation between different ethnic groups^5,16^. The frequency distribution of polymorphisms was also found to be related to clinical, epidemiological, and pathological data of patients with esophageal cancer.

Regarding smoking, no association has been found between MTHFR 677C>T polymorphisms. Concerning MTHFR 1298A>C polymorphism, this study has shown that subjects with wild-type AA genotype were more frequent among patients with esophageal cancer, either in patients who has never smoked or in active smokers, demonstrating no association between this polymorphism and the smoking habit. Limited data from few studies with small number of patients have shown no relationship between MTHFR A1298C polymorphism, smoking, and esophageal cancer, which impairs further discussion^23^.

Some studies suggested that the relationship between MTHFR C677T polymorphism and esophageal cancer could be modified by the amount of alcohol and tobacco consumption^14^. This relationship might be due to the fact that alcohol inhibits the methionine synthesis mediated by folate, which can interrupt methylation processes, inducing DNA hypomethylation in the esophageal mucosa^27^. Moreover, the alcohol has folate antagonistic effect, by reducing its absorption^14^. Conversely, a survey with 165 Japanese participants has shown no association between genotype MTHFRTT and excessive alcohol consumption^27^.

Tobacco consumption has also been shown to be responsible for the processing of vitamin B12 and folate coenzymes in a biologically inactive product^14^.

An association with MTHFR 677TT polymorphisms among drinkers and smokers and esophageal cancer has been described in previous studies^13,23^. The presence of the C allele in alcoholics and smokers was related to high risk of developing esophageal cancer^13,14,23,27^.

The conflicting results between this study and the data from the literature on the correlation of MTHFR polymorphism, alcohol and tobacco consumption, and esophageal cancer might be due to dose and frequency of exposure to these substances^13^.

Dietary factors and their effects must be considered. The low dietary folate associated with MTHFR polymorphism increases the risk of esophageal cancer. In contrast, subjects who have shown adequate serum folate levels acquired insensitivity to the effects caused by genotype MTHFR 677TT^13^.

A multiple logistic regression analysis adjusted by age, histological type, gender, ethnicity, smoking, and drinking showed that subjects with homozygous polymorphic MTHFR 677TT genotype have higher risk of death from esophageal cancer^6,12,18^. The higher risk is probably due to the following two mechanisms: changes in the normal process of methylation and unbalance levels of DNA precursors, which lead to aberrant synthesis and DNA instability^12^. Data have shown that genomic DNA methylation is lower in MTHFR 677TT genotype subjects, compared to MTHFR 677CC genotype subjects, and this difference also has had a relationship between folate levels in the bloodstream^18,14^. Higher concentrations of 5,10-methylenetetrahydrofolate resulting from MTHFR 677TT genotype may have an effect on survival of patients with esophageal cancer, because of the response of tumor cells in patients who received neoadjuvant treatment with 5-fluorouracil (5-FU)^12^. In this study, the patients who underwent neoadjuvant treatment have been excluded, so this relationship could not be evaluated. Systemic local and DNA hypomethylation has been identified in several cancer types, which induce the activation of proto-oncogenes and chromosome instability. On the other hand, hypermethylation is associated with tumor silencing suppressor gene expression. Consequently, as a key enzyme in the folate metabolism process, MTHFR may play a role in the prognosis of esophageal cancer^6,12^. This study has some limitations. This is a case-control study in which data have been retrospectively collected from medical records and questionnaires. Some variables have been reported by patients, such as ethnicity, smoking, and alcoholism. Another point to be considered is the miscegenation of the Brazilian population and the self-reported ethnicity. In this research, we did not conduct an investigation of genetic ancestry markers. In addition, this study has not evaluated the adequate intake of folate and systemic and local methylation of DNA, factors that could influence the results presented herein.

## CONCLUSION

There was no relationship between MTHFR 677C>T and MTHFR 1298A>C polymorphisms and esophageal cancer susceptibility risk. Polymorphic homozygous MTHFR677TT genotype of esophageal cancer subjects had higher risk of death from the disease after surgical treatment.
